# Plasma concentrations of secretory leukocyte protease inhibitor (SLPI) differ depending on etiology and severity in community-onset bloodstream infection

**DOI:** 10.1007/s10096-019-03567-2

**Published:** 2019-05-14

**Authors:** Anna Lange, Sara Cajander, Anders Magnuson, Jonas Sundén-Cullberg, Kristoffer Strålin, Olof Hultgren

**Affiliations:** 1 Department of Infectious Diseases, Faculty of Medicine and Health, Örebro University, SE-70182 Örebro, Sweden; 2 Clinical Epidemiology and Biostatistics, School of Medical Sciences, Örebro University, SE-70182 Örebro, Sweden; 3 Department of Infectious Diseases, Karolinska University Hospital, Stockholm, Sweden; 4 Department of Medicine Huddinge, Karolinska Institutet, Stockholm, Sweden; 5 Department of Clinical Immunology and Transfusion Medicine, Faculty of Medicine and Health, Örebro University, SE-70182 Örebro, Sweden

**Keywords:** Secretory leukocyte protease inhibitor, SLPI, Bloodstream infection, Sepsis, Sepsis immunology

## Abstract

The severity of bloodstream infections (BSI) depends on pathogen, source, and host factors. Secretory leukocyte protease inhibitor (SLPI) counteracts tissue damage, balances inflammation, and is increased in pneumonia and sepsis. We aimed to evaluate whether SLPI production differs depending on etiology, disease severity, and sex in BSI and to correlate SLPI with markers of inflammation and immunosuppression. Of the adult patients with BSI, 109 were included and sampled repeatedly, from hospital admission through day 28. Controls (blood donors) were sampled twice. SLPI in plasma was measured with enzyme-linked immunosorbent assay (ELISA) technique. *Streptococcus pneumoniae* and *Staphylococcus aureus* etiology were associated with higher SLPI than *Escherichia coli* on days 1–2 and 3. On day 1–2, subjects with sepsis had higher SLPI concentrations than those with non-septic BSI. Pneumonia was associated with higher SLPI than a non-pulmonary source of infection. SLPI co-varied with inflammatory markers. SLPI concentrations did not differ with regard to sex in the full cohort, but men with pneumonia had higher SLPI than women on day 1–2. *S. pneumoniae* and *S. aureus* BSI were associated with higher SLPI, when compared to *E. coli*. Severity and pneumonia, as well as male sex in the pneumonia sub-cohort, were factors independently associated with higher SLPI.

**Electronic supplementary material:**

The online version of this article (10.1007/s10096-019-03567-2) contains supplementary material, which is available to authorized users.

## Introduction

The clinical course of a bacterial infection is determined by the interaction of host factors, the infected organ, and the pathogen. [1]. Sepsis is defined as a life-threatening organ dysfunction caused by a dysregulated host response to infection, but there are currently no criteria for measuring the dysregulated immune response, which can manifest as disproportionate pro-inflammation and/or a state of immunosuppression [2]. The risk of death and morbidity in sepsis is still considerable [3, 4]. The causative microorganism is identified in 50–60%, and in 20–30% there is an associated bloodstream infection (BSI), defined as growth of one or more bacterial or fungal pathogens in one or more blood cultures [1, 5–7]. The reported incidence of BSI is in average 140–160/100,000/year in high-income countries, and the three most common etiologies are *Escherichia coli*, *Staphylococcus aureus*, and *Streptococcus pneumoniae* [8].

Secretory leukocyte protease inhibitor (SLPI) is a protein that has come to be seen as an important regulator of inflammation [9]. SLPI was first isolated in pulmonary secretions, and has been recognized as being not only a tissue protector that inhibits neutrophil-derived proteases but also for having other immunological tasks [10]. Similarly to antimicrobial peptides, SLPI exhibits antibacterial, antifungal, and antiviral properties, and it is known to balance pro-inflammation by downregulating the NFκB pathway [11–16]. In addition, recent research suggests that SLPI may further modulate immunity by regulating neutrophil maturation, and through inhibition of lymphocyte proliferation and the formation of neutrophil extracellular traps [17–19].

SLPI is primarily of epithelial cell origin, but is also formed by dendritic cells, macrophages, and neutrophils [11, 20–22]. The production and secretion of SLPI is regulated by pro-inflammatory stimuli [21, 23, 24].

The biological role of SLPI expression in sepsis remains only partially understood, but experimental evidence suggests that SLPI protects from detrimental inflammation. In humans, plasma SLPI has been found to be increased in sepsis, and to be associated with the degree of organ dysfunction [24].

Studies investigating SLPI in the context of sepsis are limited, and SLPI production over the course of BSI, or in relation to clinical characteristics and etiology has not been studied. We hypothesized that SLPI expression might differ depending on bacterial etiology and the source of infection. Thus, we aimed to study SLPI in a cohort of well-characterized patients with BSI followed 4 weeks. With previous findings of higher plasma SLPI concentrations in community-acquired pneumonia (CAP) in males, we also intended to study SLPI in relation to sex [25]. Finally, we wanted to see if SLPI correlates to markers of inflammation/immunosuppression.

## Methods

### Setting and study population

A prospective study of patients with BSI was conducted at Örebro University Hospital, Örebro, Sweden, between 2011 and 2014. Patients > 18 years, admitted to the Departments of Infectious Diseases and Internal Medicine, with a suspected infection, and in whom a blood culture drawn on hospital admission (day 0) showed growth of clinically significant bacteria within 3 days, were eligible for inclusion. Exclusion criteria were infection with HIV, hepatitis B and C, or previous inclusion in the study.

Blood samples were drawn from study subjects on day(s) 0, 1–2, 3, 7 ± 1, 14 ± 2, and 28 ± 4. HLA-DR expression on monocytes was measured from day 1–2. HLA-DR data is elsewhere described in detail [26]. CRP, neutrophil count, and lymphocyte count were analyzed with accredited routine laboratory methods. Patient data was obtained from medical records. Blood and plasma donors (*n* = 31, not sex- and age-matched) served as controls, and were sampled twice, 4 weeks apart. EDTA plasma was kept at − 80 °C pending analysis.

### Blood and other cultures

Two blood cultures, each consisting of 20 mL blood distributed equally between one aerobic and one anaerobic bottle, were incubated in a Bactec blood culturing system (Becton Dickinson, Franklin Lakes, NJ, USA). The bacterial species were determined by routine laboratory diagnostic procedures. Other cultures were taken depending on clinical suspicion of diagnosis according to clinical routine, and before administration of antibiotics when possible.

### Definitions

Sepsis was defined as an increase of the Sequential Organ Failure Assessment (SOFA) score by ≥ 2 points from baseline, according to Sepsis-3 definitions [2].

The diagnosis of pneumonia required a radiographic pulmonary infiltrate. Urinary tract infection (UTI) diagnosis required a urine culture testing positive for the same bacteria as the one detected in blood, and infective endocarditis was diagnosed based on the revised Duke criteria.

### ELISA analyses

SLPI in EDTA plasma was analyzed in duplicate at 1:100 dilution with Human SLPI ELISA Kit, Hycult Biotech, the Netherlands, detection range 78–5000 pg/mL, according to the manufacturer’s instructions.

### Statistics

IBM SPSS Statistics for Windows, version 24.0 (IBM Corp., Armonk, NY, USA) and STATA release 14 (StataCorp LP, College Station, TX, USA) were used. Due to non-normal distribution of SLPI, analyses were performed on log_10_ scale, or with non-parametric methods.

Patients were classified according to bacterial etiology (*E. coli*/*S. aureus*/*S. pneumonia*/other), source (pneumonia/other), and severity (sepsis/non-septic BSI). Age, sex, duration of illness, Charlson score, and severity were compared between etiology groups with chi-square, Fisher, unpaired *t*, or Mann-Whitney test, according to variable distribution.

Differences in SLPI on day 1–2 were evaluated with linear regression. The strategy was (1) unadjusted analyses of (a) bacterial etiology, (b) pneumonia/other sources, and (c) sepsis/no sepsis, in the full BSI cohort, and (d) sex in the pneumonia sub-cohort and (2) adjusted models: (a) and (b) were adjusted for age, sex, and SOFA score increase on admission, (c) for age and sex, and (d) for age and SOFA score increase.

The above analyses were performed on complete cases (*n* = 97), and, to reduce potential bias due to missing data, on all patients (*n* = 109) after multiple imputation (MI), based on Rubin’s concept [27]. Age, sex, duration of illness, SOFA score increase, bacterial etiology, immunosuppression, and Charlson score 0, 1, 2, or more were used as predictors for the SLPI imputation.

Linear mixed model with random intercept was used to evaluate mean differences of SLPI over days 1–2 to 28, for *E. coli*, *S. aureus*, and *S. pneumoniae*. Fixed factors were day, etiology, and their interaction term. The mean differences of SLPI between etiology groups on each time point were compared, and *p* values were Bonferroni-corrected. Since mixed model assumes that missing data are missing at random, demographic and clinical characteristics were compared with basic statistical methods as described above, between subjects with full and incomplete SLPI series.

Unadjusted and age- and sex-adjusted linear regression was performed to compare SLPI in BSI and controls, day 0 and 28.

Spearman correlation (*r*_*s*_) was used to correlate SLPI and inflammatory markers on day 1–2 and 7.

A *p* value < 0.05 was considered statistically significant.

## Results

### Characteristics of the study population

One-hundred-sixteen patients were enrolled. Seven were excluded due to growth of non-pathogenic bacteria (*n* = 5), or no available plasma samples (*n* = 2), leaving 109 valid subjects. Plasma was available from 46 subjects on admission, 97 (day 1–2), 68 (day 3), 86 (day 7), 78 (day 14), and 72 (day 28). Subjects with incomplete plasma series were older than those with full series (*p* = 0.02), but not otherwise significantly differing in baseline characteristics (data not shown).

Baseline and clinical characteristics and etiology are detailed in Table 1. The main sources of infection were pneumonia, UTI, skin/soft tissue, and joint infection. Fifty-four subjects had sepsis. Subjects with *S. pneumonia* BSI had higher SOFA score increases and frequency of sepsis when compared to *E. coli*. Ninety-day mortality was 10%. Eighty-nine percent of the study subjects received correct antibiotic treatment within 6 h. One subject had growth of ESBL-producing *E. coli*, and none had MRSA. Two had neutropenic sepsis (< 1000 neutrophils/mm^3^), one with polymicrobial etiology, one with beta-hemolytic streptococcal BSI.

**Table 1:** Demographic and clinical patient characteristics

Bloodstream infection etiology					
Characteristics	HC (*n* = 31)	*E. coli* (*n* = 25)	*S. aureus* (*n* = 27)	*S. pneumoniae* (*n* = 29)	Other^a^ (*n* = 28)
Age mean (SD)	52 (8)	71 (18)	72 (17)	68 (11)	68 (12)
Sex male^d^	24 (77.4)	11 (44.0)	23 (85.2)	9 (31.0)	16 (57.1)*
Number of days of disease^d^		2 (1–3)	3.5 (2–4)	2 (2–4)	1.5 (0–4)*
SOFA increase 0^d^		11 (44.0)	8 (29.6)	1 (3.4)	6 (21.4)*
SOFA increase 1^d^		8 (32.0)	6 (22.2)	7 (24.1)	8 (28.6)*
SOFA increase 2^d^		3 (12)	6 (22.2)	9 (31.0)	4 (14.3)*
SOFA increase ≥ 3^d^		3 (12)	7 (25.9)	12 41.4)	10 (35.7)*
Intensive care admission		2 (8.0)	3 (11.1)	6 (20.7)	3 (10.7)
Sepsis		6 (24.0)	13 (48)	21 (72.4)	14 (50)
Comorbid conditions					
Immunosuppression^b^		4 (16.0)	1 (3.7)	3 (10.3)	3 (10.7)
Congestive heart failure		4 (16.0)	5 (18.5)	3 (10.3)	4 (14.3)
Peripheral vascular disease		1 (4.0)	1 (3.7)	1 (3.4)	2 (7.1)
Moderate or severe kidney disease		3 (12.0)	3 (11.1)	0	2 (7.1)
Chronic liver disease		0	0	0	0
Dementia		2 (8.0)	1 (3.7)	0	2 (7.1)
Connective tissue disease		3 (12.0)	1 (3.7)	4 (13.8)	1 (3.6)
Any tumor within the last 5 years		2 (8.0)	1 (3.7)	0	2 (7.1)
Ischemic heart disease		8 (32.0)	7 (25.9)	4 (13.8)	3 (10.7)
Chronic lung disease		0	1 (3.7)	5 (17.2)	2 (7.1)
Diabetes with complications		7 (28.0)	0	0	7 (25.0)
Charlson comorbidity score		2 (0–7)	1 (0–8)	1 (0–8)	1 (0–5)
Primary focus of infection					
Lung			1 (3.7)	23 (79.3)	3 (10.7)
Airways				2 (6.9)	
Urinary tract		20 (80.0)			7 (25.0)
Skin/soft tissue			5 (18.5)		5 (17.9)
Arthritis/osteomyelitis			10 (37.0)	1 (3.4)	
Abdominal		1 (4.0)			6 (21.4)
Endocarditis		5 (18.5)			3 (10.7)
Meningitis				3 (10.3)	
Other^c^		1 (4.0)	2 (7.4)		1 (3.6)
Unknown		3 (12.0)	4 (14.8)		3 (10.7)

^a^*Betahemolytic streptocci*, *n* = 7; *Klebsiella pneumoniae*, *n* = 5,; *Polymicrobial*, *n* = 5; *Enterococci*, *n* = 4; *Pseudomonas*, *n* = 2; *Salmonella*, *n* = 2; *Streptococcus anginosus*, *n* = 1; *Haemophilus influenzae*, *n* = 1; *Actinobacillum schalii*, *n* = 1

^b^Prednisolone > 20-mg daily dose, chemotherapy, or neutropenia prior to sepsis

^c^Stent graft infection, *n* = 2; primary bloodstream infection, *n* = 2. Data is presented as *n* (%) unless otherwise stated. # median (range)

^d^Significant etiology group differences. Sex: *E. coli-S. aureus*, *p* = 0.02; *S. aureus-S. pneumoniae* < 0.01. Number of days of disease: *E. coli-S. aureus*, *p* < 0.01. SOFA score increase on admission: *E. coli-S. pneumoniae*, *p* < 0.01

### SLPI and etiology: bacteria groups and source of infection

**Fig. 1:**
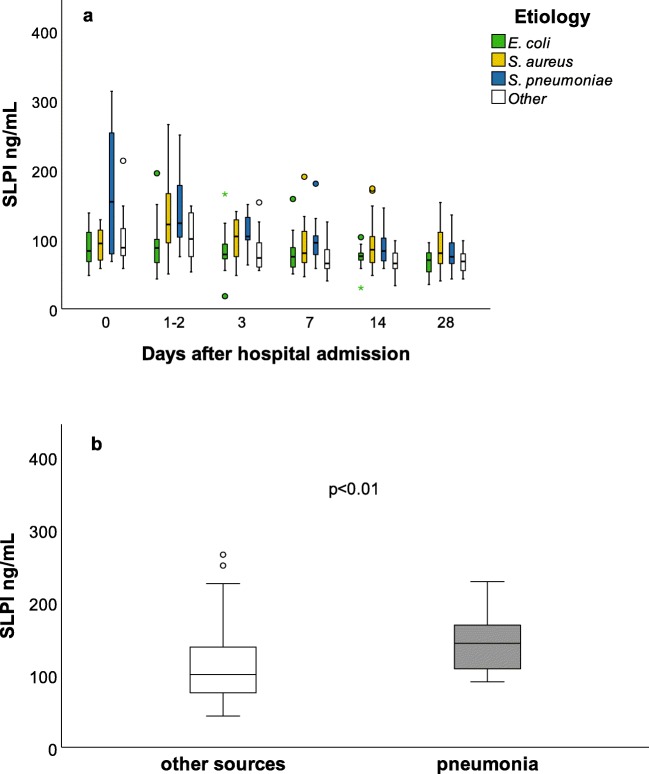
SLPI concentrations according to bacterial etiology and source of infection. **a** Bacterial etiology. The number of patients in each etiology group at specified time points is shown below the graph. We here present Bonferroni-corrected unadjusted statistically significant findings between the main bacterial etiologies: day 1–2: *E. coli-S. aureus* and *E. coli-S. pneumoniae* both *p* < 0.01. Day 3: *E. coli-S. aureus*, *p* = 0.02. *E. coli-S. pneumoniae*, *p* < 0.01. Day 14: *E. coli-S. aureus*, *p* = 0.03*.* Day 28: *E. coli-S. aureus*, *p* = 0.04. Day 1–2 statistics are also shown in Table 2. **b** SLPI concentrations on day 1–2 in pneumonia and other sources. Patients with pneumonia (*n* = 26) (median 143 ng/mL, IQR 107–170) and other sources of infection (*n* = 71) (median 100 mg/mL, IQR 75–138). The *p* value in figure is adjusted for age, sex, and SOFA score increase on hospital admission (unadjusted *p* < 0.01)

SLPI concentrations for bacterial subgroups on day 1–2 are shown in Fig. 1a. Linear regression showed a statistically significantly higher unadjusted mean SLPI concentration in *S. pneumoniae* and *S. aureus* etiology, when compared to *E. coli* in the unadjusted and adjusted (age, sex, and initial SOFA score increase) models (Table 2**)**. Analysis with MI gave the same statistically significant findings for *S. pneumoniae*. For *S. aureus* it showed *B* = 0.12, 95% CI 0.02–0.23, *p* = 0.02 in the unadjusted, and *B* = 0.08, 95% CI − 0.02–0.18, *p* = 0.1 in the adjusted model.

**Table 2:** Linear regression with log10SLPI on day 1–2 (*n* = 97) as outcome variable

	*n* (%)	Unadjusted			Adjusted for age, sex, and SOFA on admission		
		*B*	95% CI	*p*	*B*	95% CI	*p*
Age, per year		0.002	− 0.001 to 0.005	0.12	0.0009	− 0.001 to 0.003	0.43
Female sex	44 (45)	ref	ref		ref	ref	
Male sex	53 (55)	0.01	− 0.06 to 0.09	0.73	0.03	− 0.04 to 0.09	0.43
SOFA increase, per unit		0.046	0.029 to 0.062	< 0.01	0.039	0.022 to 0.056	< 0.01
*E. coli*	20 (21)	ref	ref		ref	ref	
*S. aureus*	24 (25)	0.15	0.06 to 0.25	< 0.01	0.10	0.008 to 0.20	0.04
*S. pneumoniae*	27 (28)	0.20	0.10 to 0.30	< 0.01	0.14	0.05 to 0.23	< 0.01
Other etiologies	26 (27)	0.07	− 0.02 to 0.16	0.17	0.02	− 0.07 to 0.11	0.65

Subjects with pneumonia had higher mean SLPI concentrations on day 1–2 than other sources of infection: unadjusted (*B* = 0.13, 95% CI 0.05–0.21, *p* < 0.01) and adjusted (*B* = 0.10, 95% CI 0.02–0.17, *p* = 0.01) (Fig. 1b). The same conclusion was made with MI.

### SLPI and disease severity

**Fig. 2:**
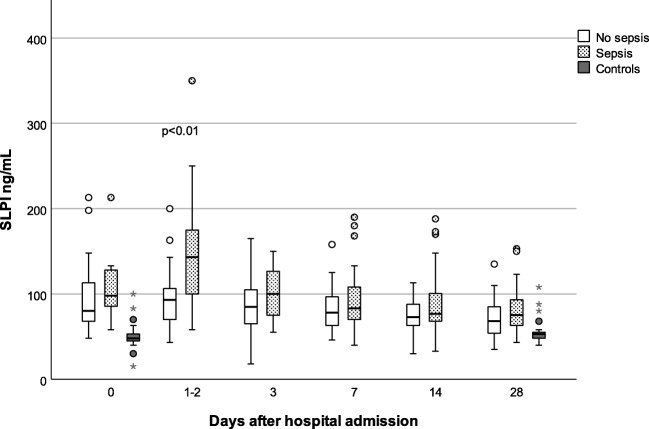
SLPI concentrations in initially septic and non-septic BSI from hospital admission to day 28 and controls day 0 and 28. Day 0: no sepsis (*n* = 26), sepsis (*n* = 20). Day 1–2: no sepsis (*n* = 48), sepsis (*n* = 49). Day 3: no sepsis (*n* = 37), sepsis (*n* = 31). Day 7: no sepsis (*n* = 43), sepsis (*n* = 43). Day 14: no sepsis (*n* = 38), sepsis (*n* = 40). Day 28: no sepsis (*n* = 39), sepsis (*n* = 33). Comparison between patients with or without sepsis is limited to day 1–2 measurements

The initial SOFA score increase was positively associated with SLPI concentrations, as shown in Table 2. Subjects with sepsis had higher mean SLPI on day 1–2 when compared to non-septic BSI: unadjusted (*B* = 0.17, 95% CI 0.10–0.23, *p* < 0.01) and adjusted (age and sex) (*B* = 0.16, 95% CI 0.10–0.23, *p* < 0.01) (Fig. 2). Analysis using MI produced similar results.

### SLPI and sex

**Fig. 3:**
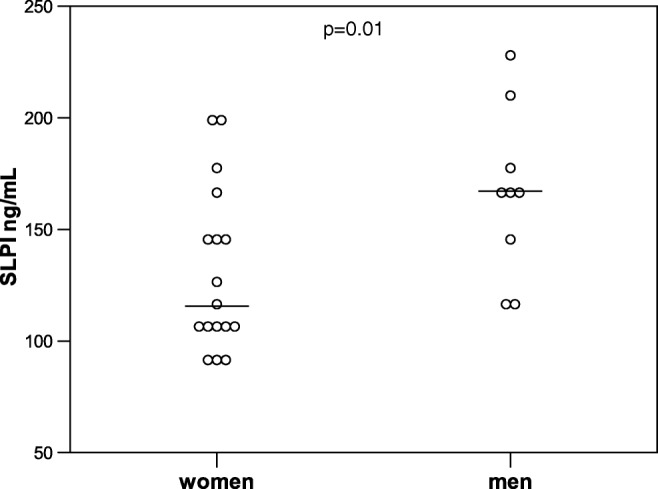
SLPI in men and women with pneumonia on day 1–2. Men (*n* = 9) median 165 ng/mL, IQR 132–193, women (*n* = 17) median 115 ng/mL, IQR 103–158. The *p* value shown in the figure is adjusted for age and severity (unadjusted *p* = 0.03). Bars represent the median

The male sex was not associated with higher SLPI in controls (*B* = − 0.001, 95% CI − 0.12–0.12, *p* = 0.98) or on day 1–2 in the full BSI cohort (*B* = − 0.05, 95% CI − 0.14–0.03, *p* = 0.2). In the pneumonia sub-cohort (men *n* = 9, women *n* = 17) however, the male sex showed statistically significantly higher mean SLPI, unadjusted (*B* = 0.11, 95% CI 0.01–0.20, *p* = 0.03), and adjusted (age and SOFA score increase) (*B* = 0.11, 95% CI 0.03–0.19, *p* = 0.01) (Fig. 3).

### SLPI dynamics

SLPI concentrations for bacterial etiology groups on days 0–28 are shown in Fig. 1. The linear mixed model interaction test between bacterial etiology and time was not significant (*p* = 0.29), showing no statistically significant different overall mean response over time. However, *S. pneumonia* etiology had higher mean SLPI concentration on day 3 (*p* = 0.01) when compared to *E. coli*, but not on day 7 (*p* = 0.06) and thereafter. *S. aureus* etiology had higher mean SLPI compared to *E. coli*, on day 3 (*p* = 0.02), day 14 (*p* = 0.03), and day 28 (*p* = 0.04), but not on day 7 (*p* = 0.36).

### SLPI in BSI and controls

Subjects with BSI had significantly higher SLPI concentrations on day 1–2 when compared to controls on day 0: unadjusted (*B* = 0.36, 95% CI 0.29–0.43, *p* < 0.01) and adjusted (age and sex) (*B* = 0.33, 95% CI 0.25–0.42, *p* < 0.01). BSI was associated with higher SLPI on day 28 in both unadjusted (*B* = 0.14, 95% CI 0.08–0.20, *p* < 0.01) and adjusted analysis (*B* = 0.10, 95% CI 0.03–0.17, *p* < 0.01) (Fig. 2).

### SLPI and other biomarkers

**Fig. 4:**
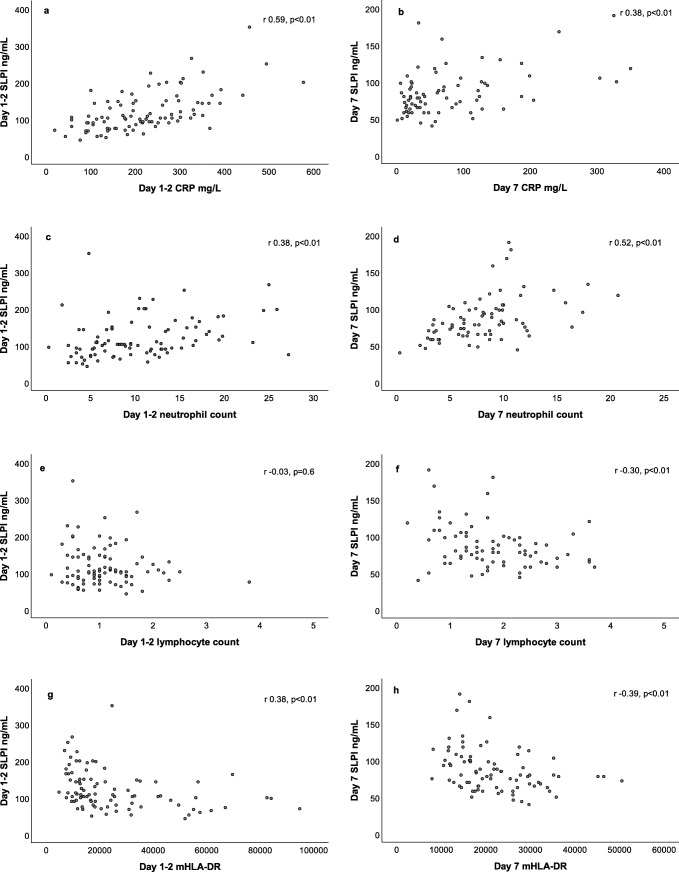
**a**–**h** Correlations between SLPI and other biomarkers on day 1–2 and day 7. SLPI-CRP on **a** day 1–2 and **b** day 7. SLPI-neutrophil count ×10^9^ on **c** day 1–2 and **d** day 7. SLPI-lymphocyte count ×10^9^ on **e** day 1–2 and **f** day 7. SLPI-monocytic HLA-DR on **g** day 1–2 and **h** day 7

SLPI did not correlate to lactate on hospital admission (*r*_*s*_ = 0.27, *p* = 0.3). SLPI was significantly positively correlated to CRP and neutrophil count, on days 1–2 and 7. It did not correlate to lymphocyte count on day 1–2 but a negative significant correlation was present on day 7. SLPI correlated negatively to HLA-DR on days 1–2 and 7 (Fig. 4a–h).

## Discussion

This study reports independent associations between bacterial etiology, disease severity, lung focus, and plasma concentration of SLPI in community-onset BSI. This is, to our knowledge, the first clinical study of SLPI as it relates to microbial etiology.

We found that, on days 1–2 and 3 after admission, subjects with *S. pneumoniae* and *S. aureus* etiology had higher plasma SLPI than *E. coli*. With account taken for the potential bias of missing samples over time, we also saw higher SLPI in *S. aureus* etiology compared to *E. coli* later in the studied period. Despite known differences in bacterial virulence, tissue tropism, and pathogen sensing by the immune system, sepsis studies rarely account for the causative pathogen [28]. We recently published another study based on this patient cohort, showing that mHLA-DR expression varies according to bacterial etiology, with low initial mHLA-DR in *S. aureus* and *S. pneumoniae* BSI [26]. A few studies have compared inflammatory markers in gram-positive and gram-negative BSI. Results are conflicting, perhaps due to study group variations. A study of general ICU patients found higher IL-6 and CRP in gram-negative sepsis [29]. Another group looked at abdominal sepsis, reporting higher levels of most pro- (TNF-α, IFN-γ, and CXCL8) and antiinflammatory (IL-1ra, IL-4, and IL-10) mediators measured, in pure gram-negative etiology. Contrastingly, a study of patients with “gram-positive cocci” (mainly *S. aureus*) and “gram-negative bacilli” (mainly *E. coli*) found no difference in IL-6 and CXCL8 concentrations [30, 31].

SLPI concentrations were associated with initial SOFA score increase, and were higher in early septic, compared to uncomplicated BSI, supporting previous results from human and animal studies. One study found an association between SLPI levels in human sepsis and the degree of organ failure [24]. Another reported increased LPS-induced immune cell activation in SLPI^−/−^ mice, more severe disease, and higher mortality in sepsis-challenged knockout mice compared to wild type (WT), and a third study correspondingly found increased LPS-induced cytokine secretion in lymph nodes in SLPI^−/−^ mice when compared to WT [22, 32].

Pulmonary sepsis is associated with high mortality [33]. We found that pneumonia was associated with higher SOFA score on admission (*p* < 0.01), than other etiologies, but adjusted analyses showed an independent association between pneumonia and SLPI, which concurs with previous reports by us and others [25, 34]. *S. pneumoniae* is the predominant pathogen in CAP, and made out 85% of BSI in our pneumonia sub-cohort. No patient had *E. coli*, and only one had *S. aureus*, which ruled out etiology-stratified analysis. SLPI is produced by epithelial cells and macrophages in the lungs, and pulmonary secretion concentrations of SLPI are increased in pneumonia [35, 36]. One study reported that, in pneumonia, SLPI in plasma was proportional to the extent of lung tissue involved, suggesting SLPI spill-over into the circulation [34]. SLPI release from specific granules upon neutrophil activation might also contribute to increased plasma SLPI [37]. Additionally, SLPI is secreted by other epithelial cells, and another source of plasma SLPI could be protein leakage from other epithelial sites than the lungs [9]. A study of acetaminophen-induced acute liver failure exemplified this, showing that circulating SLPI derived from the liver [38]. It is yet possible that epithelial affection is more significant in pneumonia, explaining some of the observed differences.

Despite the low number of study subjects, and in line with our previous findings with a larger cohort of CAP, we report higher plasma SLPI, independently of severity, in men than in women with pneumonia and BSI on day 1–2 [25]. Due to loss of sampling over time, we could not assess the dynamics of this sex-related difference. Through unknown mechanisms, the sexes display differences in infection and immunity already in childhood. Females are predisposed to autoimmunity and better vaccine responses, whereas males are more susceptible to some infections [39, 40]. In terms of sepsis, a male preponderance has been demonstrated in both adults and children, and as to pneumonia, male sex is a risk factor for severity [1, 7, 41, 42]. Experimental studies indicate that female sex hormones are protective in sepsis [43]. Interestingly, sex hormones, or sex hormone receptors, have been suggested to be involved in the regulation of SLPI expression [44–46].

SLPI covaried with CRP and neutrophil count, and remained elevated when compared to controls throughout this study. The biological significance of circulating SLPI is unknown, but SLPI was detected in the nucleus of peripheral blood monocytes in sepsis patients, and it was shown in vitro that monocytes and B cells internalize exogenous SLPI [16, 47]. SLPI does not influence mHLA-DR expression in vitro, but the observed inverse correlation between these two markers might suggest that SLPI in plasma does not only reflect a pro-inflammatory state or tissue damage, but is possibly linked to sepsis-induced immunosuppression [38]. Future studies might identify whether circulating SLPI penetrates immune cells to antagonize inflammatory responses, and if the correlation between SLPI and disease severity, as demonstrated by us and others, relates to that.

This study has some limitations, including the limited number of subjects, half of them not sampled for plasma on admission, making the study underpowered for etiological comparison before antibiotic were given. Secondly, missing data may affect the validity of the over-time analyses, and equivalently, MI analysis showed lower association for *S. aureus* etiology; hence, these results are interpreted with caution. Furthermore, Sweden has a low degree of antibiotic resistance compared to most countries, and the greater part of subjects with *E. coli* BSI had a UTI, which is known to be associated with milder disease than an abdominal focus [48]. Therefore, in other settings, conclusions might have been different.

Strengths of this study include clear definitions of BSI and etiology, and varying disease severity, reflecting the heterogeneity of BSI and sepsis. Development of treatments targeting the dysregulated host response in sepsis will require deeper knowledge in how host-related factors, e.g., sex, and specific pathogens, influence the immune response.

In summary, this study shows differences in SLPI blood concentrations related to bacterial etiology, disease severity, and source in BSI, plus a sex-related divergence in SLPI concentrations in pneumonia. SLPI is noticed for versatile immunological functions, and our results warrant further studies that elucidate the role of SLPI in severe bacterial infections.

## Electronic supplementary material

ESM 1 (XLSX 39 kb)
